# DOES THE MICROBIOME INFLUENCE THE DEVELOPMENT OF DEMENTIA IN OLDER ADULTS?

**DOI:** 10.1093/geroni/igac059.1812

**Published:** 2022-12-20

**Authors:** Naomi Tesema, Kristen Wroblewski, Jayant Pinto, Martha McClintock

**Affiliations:** University of Chicago, Chicago, Illinois, United States; University of Chicago, Chicago, Illinois, United States; University of Chicago, Chicago, Illinois, United States; University of Chicago, Chicago, Illinois, United States

## Abstract

Some data implicate microbes in the pathogenesis of Alzheimer’s Disease and Related Dementias (ADRD). Whether the use of antibiotics predisposes to ADRD remains unknown. To investigate the relationship between antibiotics and subsequent cognitive function, we analyzed data from a longitudinal, nationally representative sample of older US adults (N=2,906, the National Social Life, Health and Aging Project). The use of antibiotic medications was collected by home interview at baseline. Five years later, cognition was assessed using a survey adapted version of the Montreal Cognitive Assessment (MoCA). Additionally, ADRD status was measured by a report of a physician’s diagnosis (self or proxy). The association between baseline antibiotic use and an interval dementia diagnosis/MoCA scores was tested using multivariate logistic regression, controlling for age, sex, race and ethnicity, education, co-morbidities (modified Charlson Comorbidity Index), and cognition at baseline (Short Portable Mental Status Questionnaire). Older US adults who used antibiotics had lower MoCA-SA scores at 5-year follow-up (OR=3.94, 95% CI= 1.79-8.66). However, the use of antibiotics did not predict a subsequent diagnosis of dementia (OR=1.48, 95% CI= 0.44-4.95). Thus, antibiotic use may cause deleterious effects on cognitive function, but does not appear to have a clinical impact in terms of diagnosis of dementia. Further study of the role of microbes and drugs that modulate them may be useful in understanding ADRD.

